# Alterations in the Hippo Signaling Pathway During Adenogenesis Impairment in Postnatal Mouse Uterus

**DOI:** 10.1007/s43032-025-01793-y

**Published:** 2025-02-11

**Authors:** İrem İnanç, Onur Bender, Arzu Atalay, Serdal Kenan Köse, Esra Erdemli

**Affiliations:** 1 Faculty of Medicine, Department of Histology and Embryology, Ankara University, Ankara, Turkey; 2 Biotechnology Institute, Ankara University, Ankara, Turkey; 3 Faculty of Medicine, Department of Biostatistics, Ankara University, Ankara, Turkey

**Keywords:** Uterine gland, Adenogenesis, Hippo signaling pathway, YAP, p-YAP

## Abstract

The mouse uterus, which consists of single-layered epithelium and undifferentiated mesenchyme at birth, begins to differentiate in the postnatal period. The process of adenogenesis, defined as gland development, begins on the Postnatal (PN) Day 5, and this process is very evident on the PN Day 10. Although various signaling pathways effective in the adenogenesis process but the mechanism underlying this progress have not been clarified yet. Hippo signaling pathway have roles in many cellular functions, such as proliferation, differentiation and cell death. But the relationship between the Hippo signaling pathway and uterus adenogenesis is unknown. The objective of this study has been to determine if there is a change in the Hippo signaling pathway in mice with impaired gland development during the adenogenesis process. To that aim, we use mouse uterus with normal gland development (control group) and gland development inhibited by progesterone (experimental group). Animals were sacrificed on the PN Days 5, 10 and 15. YAP and p-YAP by immunohistochemistry and immunoblotting techniques to identify the main components of Hippo Signaling Pathway. *YAP*, *LATS1*, *LATS 2*, *MST1*, *NF2* and *TAZ* used for the RT-qPCR methods. In conclusion, Hippo signaling pathway components were reduced during the adenogenesis process in mouse with impaired gland development.

**Graphical Abstract:**

A summary of our study. When the control and experimental groups were compared, significant differences were observed in terms of the Hippo signaling pathway.

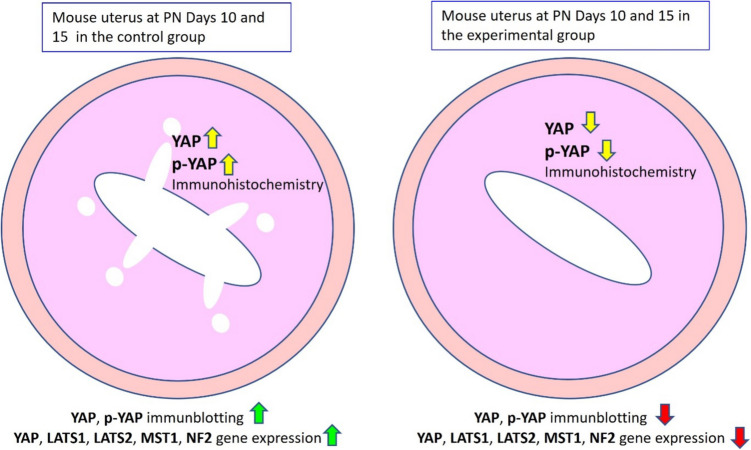

**Supplementary Information:**

The online version contains supplementary material available at 10.1007/s43032-025-01793-y.

## Introduction

Although many reproductive organs complete their development and differentiation in the fetal period, the uterus fulfils this process in the postnatal(PN) period. The mammalian uterus is surrounded by surface epithelium and an undifferentiated mesenchyme at birth [1]. Adenogenesis, which is characterized as the formation of uterine glands budding from the luminal epithelium, begins predominantly in the PN period in domestic and laboratory animals [2, 3] and then these glands extend along the stroma and become convoluted [4]. On PN Day 5, invaginations begin from the epithelial lumen to the stroma [5]. PN Day 10 is critical for the adenogenesis process and uterine gland development. The uterus shows adult morphology features of PN 15–20 days [6] [Figure S1(A)]. However, the adenogenesis in humans begins from fetal life and ends in the puberty period [7].

Changes in uterine morphogenesis after birth are a very complex process [8]. The stroma-derived growth factors play an important role in uterine morphogenesis, such as proliferation, differentiation and the branching process of the epithelium [9–12]. FOXA2 (Forkhead box protein A2) [13, 14], Wnt signaling pathway(Wingless and Int-1) [8, 15], CDH1(Cadherin 1) [16], TGF-β (Transforming growth factor-beta), Homeobox10, 11, insulin growth factors [17] and CK1α (Casein kinase 1 alpha) [7] have been stated as among the factors affecting adenogenesis. It is very important for the glandular epithelium to develop through the proliferation that occurs in the epithelium during the adenogenesis process [5] but the pathways regulating adenogenesis have not been fully elucidated [2].

The Hippo signaling pathway, which was discovered for the first time in *Drosophila* and also preserved in mammals, is known to take part in many cellular functions, such as proliferation, differentiation and cell death [18]. The downstream effectors of the Hippo signaling pathway operating as a kinase cascade in mammals are MST1/2 (mammalian STE20-like protein kinase 1 and 2) and LATS1/2 (large tumour suppressor homologous kinase 1 and 2). Its transcriptional coactivators are YAP (yes-associated protein) and TAZ (paralog of YAP, its PDZ-linked motif-containing transcriptional coactivator, also known as WWTR1). While the Hippo signaling pathway is active, MST1/2, LATS1/2 and YAP/TAZ are phosphorylated in the cell cytoplasm. Depending on the YAP/TAZ phosphorylation, it either remains in the cytoplasm by binding to the 14-3-3 protein or is degraded in the proteosome. While the Hippo signaling pathway is inactive, the YAP is not phosphorylated and clusters in the nucleus and regulates various transcriptional genes involved in cell growth, survival and proliferation by binding the TEAD domain of the nucleus [18–20]. In addition to these components that make up the Hippo signaling pathway, there are also coactivators MOB1 (Mps-1 binding) that bind to Sav1 (Salvador 1) and LATS1/2 kinases that bind to MST1/2 [21] [Figure S1(B)]. It has been revealed that many internal and external factors, such as cell-cell junctions, polarisation, stress and energy states, can determine whether the Hippo signaling pathway is active or not [22].

It is also known that secretions of the endometrial glands are required for the placenta as a food source for the successful implantation of the embryo [23–25]. It has been considered that the reason for the high rates of undetermined embryo loss in the peri-implantation period may be due to errors in endometrial gland morphogenesis [2]. Although the Hippo signaling pathway and uterine morphogenesis are both active in many processes, such as proliferation and differentiation of cells, these two concepts have not been fully elucidated. For this reason, the aim of our study was to determine whether the Hippo signaling pathway is effective in uterine gland development in the PN period. This study is descriptive, both in explaining the signal pathways that may be effective in the changes that occur in the PN period of the uterus and illuminating the causes of infertility.

## Materials and Methods

### Animals and Treatments

In total, 30 wild type *Balb/c* female mice were purchased from the Experimental Animal Facility of Ankara University. All procedures were conducted in accordance with Ankara University Animal Experiments Ethics Committee approval No. 2020-21-168.

Our work was based on the study of Filant et al. [26] to prevent the development of uterine glands in the PN period. For this purpose, 50 µg/g progesterone(P4) (Sigma Aldrich P0130) was dissolved in 0.1 ml sesame oil (Sigma Aldrich-S3547). This solution was administered as a subcutaneous injection every day to prevent gland development between PN Days 2 and 10. For the control group, 0.1 ml sesame oil was administered as a subcutaneous injection every day between PN Days 2 and 10. In order to observe the stages of uterine morphogenesis, the control (only sesame oil given) and the experimental group (sesame oil and progesterone given) animals were sacrificed by cervical dislocation on PN Days 5, 10 and 15 (Figure S2). For each day, 5 animals were used in the control and experimental groups. The removed tissues were placed in buffered formalin for hematoxylin and eosin staining and immunohistochemistry analysis. For electron microscopy, the tissues were placed in buffered glutaraldehyde. For western blot and gene analysis, samples were stored at − 80 °C.

### Tissue Processing

The uterus samples were fixed in 10% buffered formalin solution for 48–72 h. They passed through tap water and then through an increased series of alcohols. Tissues cleared in xylene were kept in hot paraffin at 60 °C, and afterwards, paraffin blocks were formed. Sections of 4-µm thickness were taken from paraffin blocks with a Leica RM 2125RT model microtome.

### Hematoxylin and Eosin Staining

After keeping the sections in an incubator at 60 °C for 1 h, they were exposed to xylene twice for 30 min for deparaffinisation. The sections were passed through 100%, 96% and 75% ethanol series, respectively, for rehydration and were then washed with tap water. After staining with a hematoxylin solution for 1 min and with eosin solution for 3 min, the sections were washed in tap water. For the dehydration process, the sections were passed through 75%, 96% and 100% ethanol series. For transparency, the sections were passed through xylene twice for 30 min and covered with a coverslip using Entellan. The stained preparations were examined with a Zeiss Axio Scope A1 (Carl Zeiss, Oberkochen, Germany) light microscope and photographed.

### Electron Microscopy

The uterus samples were fixed with 2.5% glutaraldehyde in 0.1 M phosphate buffer (pH 7.4) at room temperature (RT) for 1 h and postfixed in 1% phosphate-buffered osmium tetroxide for 1 h. The samples were dehydrated by passage through a graded ethanol series and embedded in Araldite. Ultrathin sections were contrast stained with uranyl acetate and lead citrate and observed under a HITACHI HT7800 transmission electron microscope.

### Immunohistochemistry

Sections of 4-µm thickness were taken from paraffin blocks on poly-L-lysine-coated slides. After being deparaffinised with xylene(Sigma Aldrich, 108298), the sections hydrated by passing through 100%, 90%, 80% and 70% grade alcohol series. After inhibition of antigenic masking with citrate buffer (Ph 6) (Sigma Aldrich, W302600), the sections were washed with phosphate-buffered saline (PBS). Sections were treated with 0.3% H_2_O_2_(Thermo Scientific, TA 060-HP) to prevent endogenous peroxidase activity. After washing the sections with PBS, they were incubated for 5 min at room temperature with blocking solution (Thermo Scientific, TA-060-UB). Primary antibodies were prepared in appropriate dilutions(YAP 1:400 [Cell Signaling Technology], p-YAP 1:1250 [Cell Signaling Technology] and FOXA2 1:200 [Bioss]) applied to the sections overnight at + 4 °C. For negative controls, isotype IgG(Jackson Immuno Research) was applied.

After incubation with primary antibody, sections were washed with PBS and incubated with biotinylated secondary antibody (Thermo Scientific, TL-060-PB) for 10 min at room temperature. The sections were washed with PBS and incubated with streptavidin peroxidase (Thermo Scientific TL-060-PH) complex for 10 min. The sections were washed with PBS and treated with 3,3-diaminobenzidine (DAB) solution as a chromogen. By reverse staining with Harris Hematoxylin (Sigma Aldrich, HHS16), the sections were dehydrated by passing 70%, 80%, 90% and 100% alcohol series. The sections were held in xylene twice for 30 min and then covered with Entellan (Merck, 13073-00). The stained preparations were examined with a Zeiss Axio Scope A1 (Carl Zeiss, Oberkochen, Germany) light microscope.

Cells showing immunoreactivity in the uterus were evaluated using ImageJ software (ImageJ1,51j8, National Institutes of Health, USA). These cells were evaluated with H-SCORE, as described before [27]. The evaluation was conducted by two blinded observers, and an H-SCORE value was derived by summing the percentages of cells that stained at each intensity multiplied by the weighted intensity of the staining (H-SCORE = Σ·Pi [i + 1] for each tissue, where i is the intensity score and Pi is the corresponding percentage of the cells).

GraphPad Prism version 10.0 and unpaired two tailed Student t-test used for statistical analysis for immunohistochemistry analyses, and p values less than 0.05 were considered statistically significant.

### Protein Isolation and Western Blot

Western blot analyses were performed following the protocols we previously described [28].Uterus samples were homogenized using the TissueLyser LT system (Qiagen, Hilden, Germany) with a 5-mm stainless steel bead (Qiagen) in a T-PER Tissue Protein Extraction Reagent (Thermo Fisher Scientific, Waltham, MA, USA) containing protease and phosphatase inhibitor cocktails. Homogenization was carried out by mechanical disruption of the ice-cold samples at 50 Hz for 3 min. Afterwards, the samples were immediately centrifuged at 10,000 g for 6 min at 4 °C. Supernatants were collected and quantification of proteins was performed with the TaKaRa BCA Protein Assay Kit (Takara Bio, Shiga, Japan) according to the manufacturer’s instructions. Proteins were diluted with Laemmli 4X buffer (Bio-Rad, Hercules, CA, USA) with 5% 2-mercaptoethanol (Bio-Rad) as a denaturing agent and boiled at 95 °C for 6 min. For each sample, 10 µg of protein was loaded into the wells of an Any kD Mini-PROTEAN TGX Stain-Free Protein Gels (Bio-Rad). Running was performed at 100 V with the Mini-PROTEAN Tetra Vertical Electrophoresis Cell System (Bio-Rad) using Tris/Glycine/SDS Electrophoresis Buffer (Bio-Rad). Proteins were then transferred from the gel onto an Immobilon-P PVDF Membrane (Merck Millipore, Burlington, MA, USA) with a Mini Trans-Blot Module (Bio-Rad) for 60 min. Membranes were blocked with 5% BSA (Cell Signaling Technology, Danvers, MA, USA) solution in TBS-T for 60 min and then labelled with primary antibodies overnight at 4 °C. The primary and secondary antibodies were listed as follows: YAP (Cell Signaling Technology, 14074), p-YAP (Cell Signaling Technology, 13008) and Beta Actin (Bioss Antibodies, bs-0061R). YAP and p-YAP antibodies were used at 1/1,000 dilution and Beta Actin at 1/5,000 dilution. After serial washing, the membranes were treated with anti-rabbit IgG, HRP-linked secondary antibody (Cell Signaling Technology, 7074) for 1 h at RT. After a final series of washes, the membranes were labelled with Clarity Western ECL Substrate (Bio-Rad) and chemiluminescence imaging was performed on the Odyssey^®^ XF Imaging System (LI-COR Biosciences, Lincoln, Nebraska, USA) [29].

### RNA Isolation and cDNA Synthesis

Total RNA isolation from the uterine tissues was performed with the miRNeasy Mini Kit (Qiagen, Hilden, Germany). The uterine tissues were first mechanically disrupted and homogenized using the TissueLyser LT system (Qiagen). Each tissue sample was placed in 2 mL of a Safe-Lock tube with a 5 mm stainless steel bead (Qiagen) and 700 µl of QIAzol Lysis Reagent and incubated on ice for 10 min. It was then homogenised for 3 min at 50 Hz and incubated for 5 min at room temperature. Next, 140 µl of chloroform was added to the lysate and centrifuged at 12,000 g for 15 min at 4 °C. The upper aqueous phase was transferred to the spin column and the next steps of RNA isolation were followed according to the miRNeasy Mini Kit protocol. RNA quantity and purity were measured using a NanoDrop 2000 spectrophotometer (Thermo Fisher Scientific, Waltham, MA, USA). RNAs were reverse transcribed with an iScript cDNA Synthesis Kit (Bio-Rad, Hercules, CA, USA) according to the manufacturer’s instructions. The cDNA reaction mix with 1 µg RNA template was incubated at 25 °C for 5 min, 46 °C for 20 min and 95 °C for 1 min in a T100 thermal cycler (Bio-Rad, Hercules, USA).

### Quantitative Real-Time RT-PCR

Quantitative real-time PCR analysis was utilised with QuantiTect SYBR Green PCR Master Mix (Qiagen, Hilden, Germany), which contains HotStarTaq DNA Polymerase, QuantiTect SYBR Green PCR Buffer, and dNTP mix including dUTP, SYBR Green I and 5 mM MgCl_2_ on LightCycler 480 system (Roche, Mannheim, Germany). The amplification protocol consisted of an initial denaturation step at 95 °C for 15 min, followed by amplification for 40 cycles, each cycle consisting of denaturation at 94 °C for 15 s, annealing at 60 °C for 30 s and extension at 72 °C for 30 s. The primer sequences, template accession IDs and amplicon lengths are given in Table 1. The results were analysed using the absolute quantification method with known standards on LightCycler 480 Software v1.5.0.39. Amplification of GAPDH, a housekeeping gene, was used to normalise the expression levels. Statistical analysis was performed using unpaired two-tailed Student t-test via GraphPad Prism for quantitative real-time PCR studies. Statistically significiant results were represented as follows: *, *p* < 0.05.

**Table 1:** Primer sequences, template accession IDs and amplicon lengths of target genes

Target	Primer sequences	Template accession ID	Amplicon length (bp)
YAP	F: 5’- CCCTCGTTTTGCCATGAACC − 3’ R: 5’- ATTCCGTATTGCCTGCCGAA − 3’	NM_001171147.1	205
LATS1	F: 5’- GAGTTACCAAGACCCTCGTCG − 3’ R: 5’- TCTGTCCATTGCTTGGGTGA − 3’	NM_010690.1	275
LATS2	F: 5’- GTTTCCAACTGTCGCTGTGG − 3’ R: 5’- GCCCAACCAGCATCTCAAAG − 3’	NM_015771.2	196
MST1	F: 5’- TCCTACAGCACCCGTTTGTT − 3’ R: 5’- GTATTGGCTCCTCCGCTCAT − 3’	NM_021420.4	235
NF2	F: 5’- GCTCAAGACGGAGATCGAGG − 3’ R: 5’- CTGCAGAGTGAGTTTGAGGACT − 3’	NM_001252250.1	175
TAZ	F: 5’- CCTTATCACCGTCTCCAACCAC − 3’ R: 5’- CCTTGGTGAAGCAGATGTCTGC − 3’	NM_001173547.2	134
GAPDH	F: 5’- CAGGAGAGTGTTTCCTCGTCC − 3’ R: 5’- GATGGGCTTCCCGTTGATGA − 3’	NM_001289726.1	247

## Results

### Morphological Investigation of Uterine Gland Development by Hematoxylin and Eosin Staining, Immunohistochemistry Staining of FOXA2 and Electron Microscopy Analysis

**Fig. 1:**
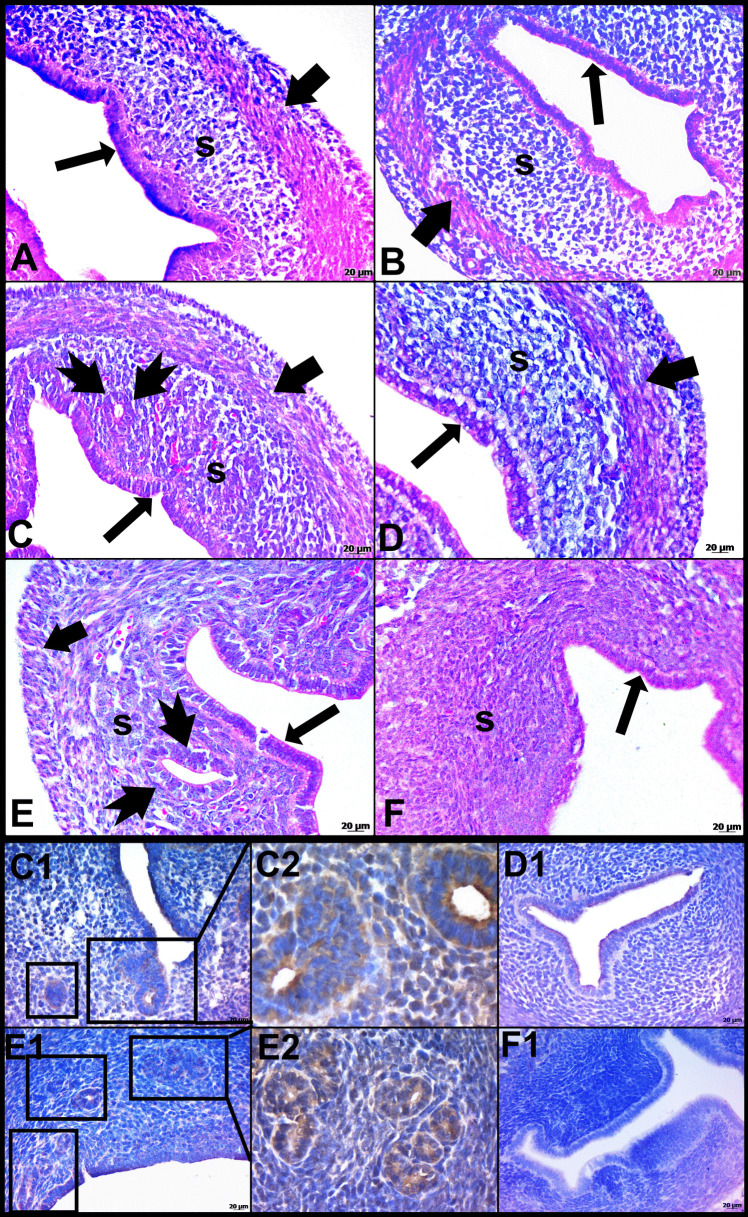
Hematoxylin and eosin staining of control (**A**,** C**,** E**) and experimental groups (**B**,** D**,** F**). Immunohistochemistry staining of FOXA2 shown in **C1-F1**. On PN Day 10 and 15, the uterine glands were evident in the control group, but no glands were observed in the experimental group. The control and experimental groups showed similar characteristics on PN Day 5. While FOXA2 staining was observed in the glands of the control groups on PN Days 10 and 15, no similar staining determined in the experimental groups. **A-B**: Postnatal 5 Day, **C-D**: Postnatal 10 day, **E-F**: Postnatal 15 day, **C1-C2**: Postnatal 10 day of control group, **D1**: Postnatal 10 day of experimental group, **E1-E2**: Postnatal 15 day of control group, **F1**: Postnatal 15 day of experimental group. **C2**, **E2**: Closer view of the glands. **⬆**: epithelium;➨: muscle tissue; **tailed arrow**: glands; **s**: stroma; **□**: glands

On PN Day 5, uterine gland invaginations from the epithelium to the stroma started to develop. On PN Day 10, it was quite evident that these invaginations towards the stroma extended and formed the glandular structure. At the same time, it is noteworthy that the muscle layer is more organized. On PN Day 15, it was determined that the uterine architecture was fully developed. Accordingly, there was a columnar epithelium surrounding the uterine lumen, in the stroma the uterine glands, and inner circular and outer longitudinal smooth muscle bundles surrounding the stroma were evident (Fig. 1).

The control and experimental groups showed similar characteristics on PN Day 5. In addition, the uterine lumen is surrounded by columnar epithelium and there was a prominent stroma around it. The myometrium was also visible. On PN Day 10, the uterine glands were evident in the control group, but no glands were observed in the stroma of the experimental group. The epithelium and myometrium showed similar features in both groups. On PN Day 15, there were no glands in the stroma in the experimental groups compared to the control group. The epithelium and myometrium were similarly observed in both groups(Fig. 1). Since there was a difference in gland development between the control and experimental groups on PN Day 10, the gland-specific marker, FOXA2, used by immunohistochemical method. On the PN Days 10 and 15, a significant staining with FOXA2 was observed in the stroma of the control groups glands. Because of the gland development was inhibited in the experimental groups, no staining with FOXA2 was determined in the stroma (Fig. 1).

**Fig. 2:**
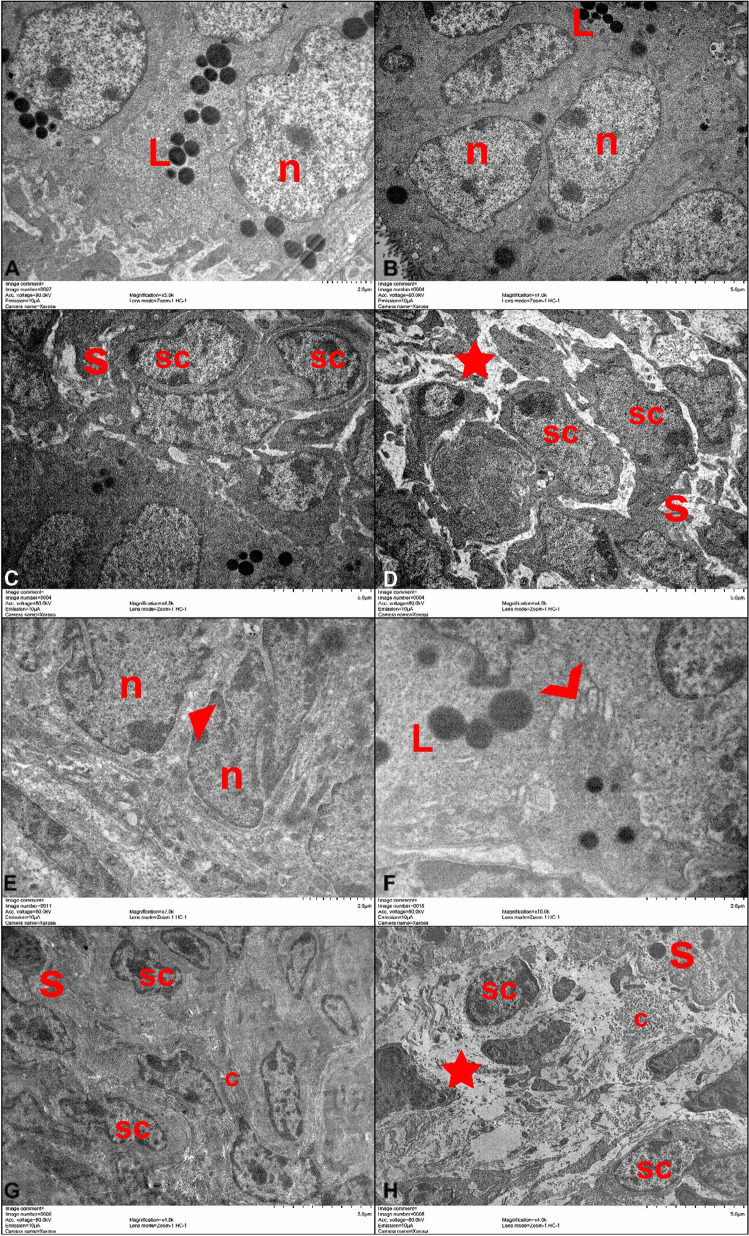
Electron microscopy analysis of the groups. When compared to the contol group, gaps were observed between the epithelium and the stroma was edematous in the experimental group. **A**,** C**,** E**,** G**: Control Groups; **B**,** A**,** F**,** H**: Experimental Groups. **A-D**: Postnatal 10 Day, **E-H**: Postnatal 15 Day. **n**: nucleus in the epithelium ; **L**:lipid droplets; **s**: stroma; **sc**: stromal cell nucleus ★:edema **c**:collagen; ◣: normal gaps; ❯: wide gaps

In our electron microscopy analysis, on PN Day 10, the epithelium showed similar characteristics in the control and experimental groups. The euchromatin nuclei of the cells showed analogous features. Lipid droplets were seen in both groups and had an similar structure. The experimental groups and the control groups showed differences only in the stroma. Especially the edematous areas in the experimental groups were remarkable. In the control groups, the cells were arranged closer to each other in the stroma. On the PN Day 15, the epithelium showed similar characteristics of nucleus and lipid droplets in both groups. However, the wide gaps were observed at the base of the epithelium in the experimental groups. The connection units of the control groups epithelium were normal. Collagen bundles were also observed in the stroma in both groups. In the control group, the cells in the stroma were in close contact with each other. But in the experimental group, edematous areas were also evident in the stroma. When we compared the groups, edematous regions in the stroma were more enlarged on the PN Day 15 (Fig. 2).

### Immunohistochemistry Analysis of YAP and p-YAP in Adenogenesis Process

**Fig. 3:**
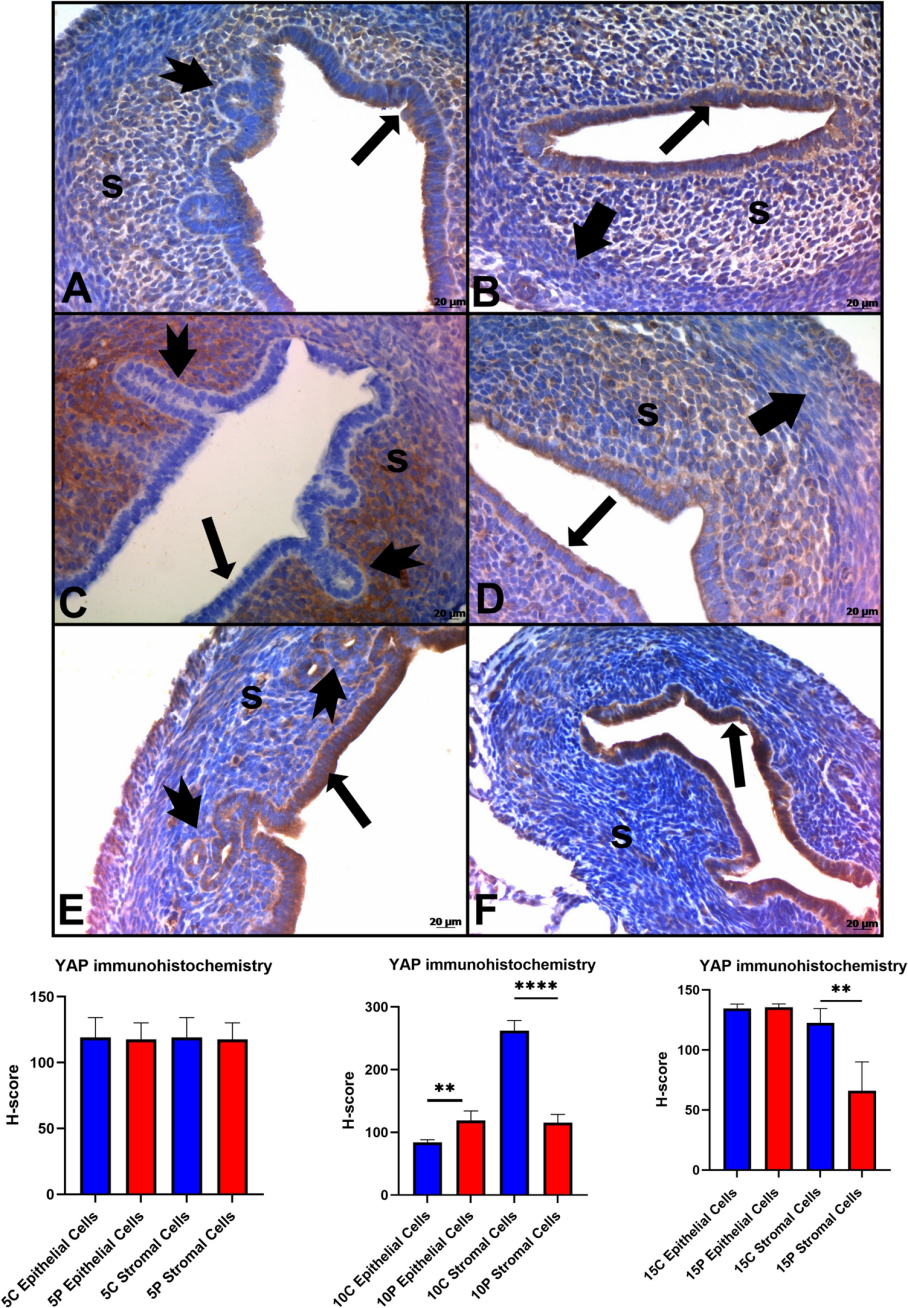
Immunohistochemistry staining and H-Score analysis of YAP. YAP expression was weaker in the stroma of the experimental group on PN 10 and 15 days. **A**,** C**,** E**: Control Groups, **B**,** D**,** E**: Experimantal Groups. **A-B**: Postnatal 5 Day, **C-D**: Postnatal 10 day, **E-F**: Postnatal 15 day **⬆**: epithelium; ➨: muscle tissue; ** tailed arrow**: glands; **s**: stroma

On PN Day 5, the expression of YAP in the control and experimental groups showed similar features. This expression was mostly observed in the cytoplasm of the columnar epithelium and in the stroma cells. It was observed that the expression of YAP was quite intense in the control group, especially on PN Day 10. Expression was determined in the cytoplasm of the cells and in the nuclei of some cells in the stroma. On PN Day 10, no glandular structure was observed in the stroma in the experimental group. It was observed that the expression of YAP decreased in the stromal cells but increased in epithelial cells on PN Day 10 compared to the control group(*p* < 0.05). On PN Day 15, YAP expression was weaker in the stroma of the experimental group than the control group (*p* < 0.05) (Fig. 3). Negative control stainings of YAP were shown in Figure S3.

**Fig. 4:**
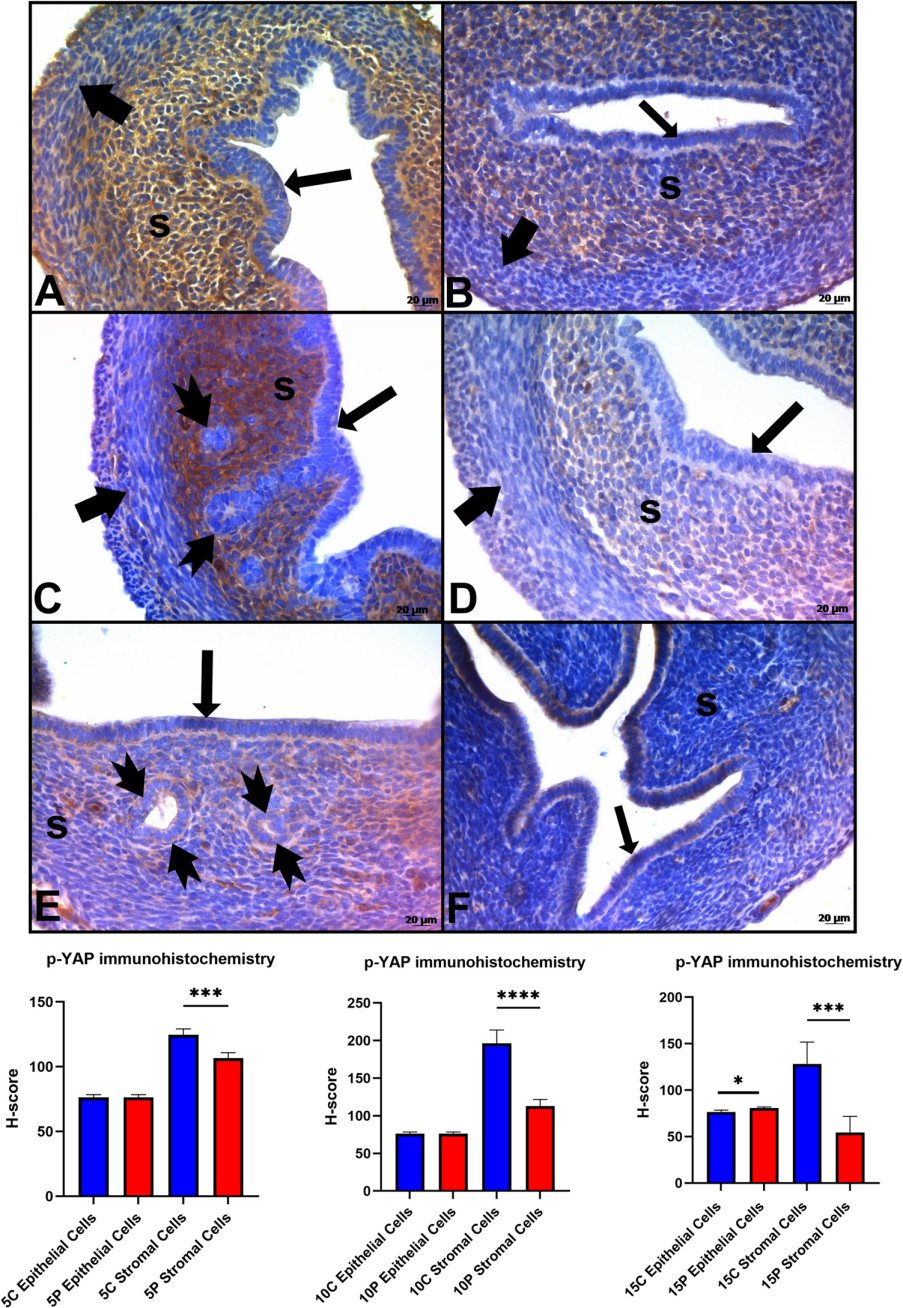
Immunohistochemistry staining and H-Score analysis of p-YAP. **A**,** C**,** E**: Control Groups, **B**,** D**,** E**: Experimantal Groups. **A-B**: Postnatal 5 Day, **C-D**: Postnatal 10 day, **E-F**: Postnatal 15 day **⬆**: epithelium;➨: muscle tissue; **tailed arrow**: glands; **s**: stroma

On PN Day 5, p-YAP expression was shown higher expression in the stroma cells in the control group (*p* < 0.05). When compared to the control group, p-YAP expression, which was determined in the stroma cells, was quite low in the experimental group on PN Day 10 (*p* < 0.05). On PN Day 15, p-YAP expression showed a difference between the control and experimental groups (*p* < 0.05). In the experimental groups the epithelial cells and in the control groups the stromal cells of p-YAP expression was higher (*p* < 0.05)(Fig. 4). It was also observed that the expression of p-YAP in the control group on PN Day 15 decreased compared to PN Day 10. Negative control stainings of p-YAP were shown in Figure S4.

### Differential YAP and p-YAP Activity in Postpartum Period

**Fig. 5:**
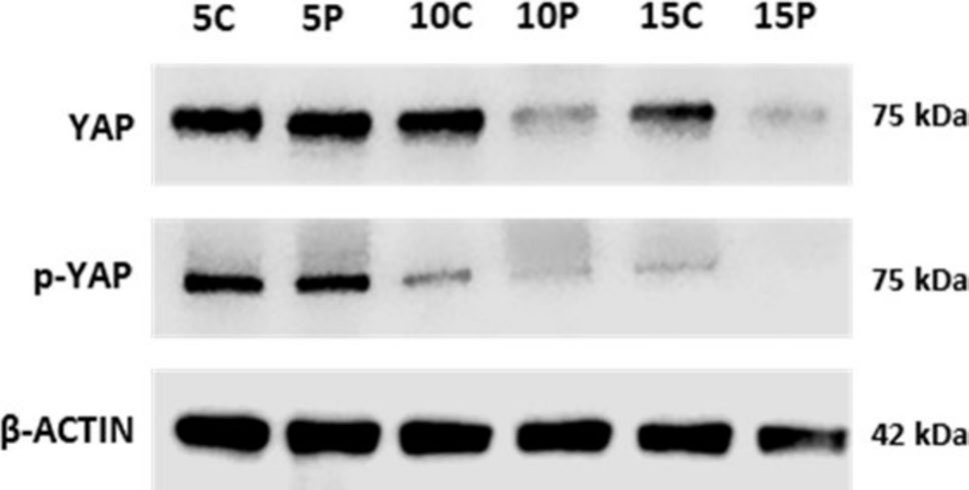
Protein analysis of YAP and p-YAP. **C**:Control Groups, **P**: Progesterone administered Experimantal Groups. **5**: Postnatal 5 Day,**10**: Postnatal 10 day, **15**: Postnatal 15 day

Total YAP levels and its phosphorylation were evaluated by monitoring YAP activity in the control and experimental groups in the postpartum period. Figure 5 shows the expression of YAP and p-YAP in the control and experimental groups on PN Day 5. While high levels of YAP expression were observed in the control group on PN Days 10 and 15, this expression was considerably reduced in the experimental group. The expression of p-YAP on PN Day 10 was quite low in the experimental groups when compared to the control group. On Day 15, less expression was observed in the control group compared to the previous days, and no expression was observed in the experimental group (Fig. 5).

### mRNA Expression Patterns of Hippo Signaling Pathway During Adenogenesis

**Fig. 6:**
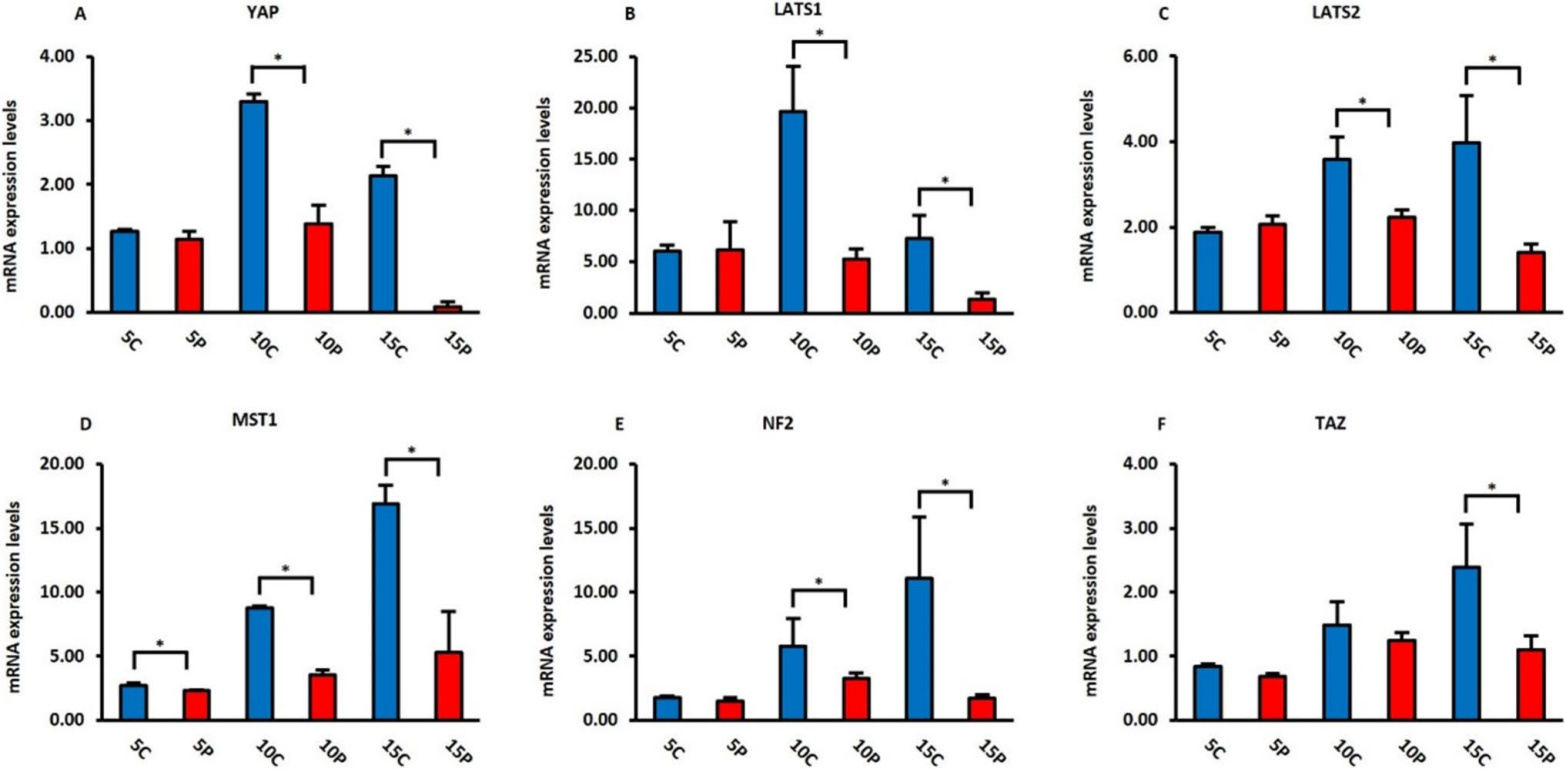
Quantitative real-time PCR analysis of control and experimental groups of mouse uterus. **C**: Control; **P**: Progesterone administered experimental group

In the PN mouse uterus, mRNA expression changes in *YAP*, *LATS1*, *LATS2*, *MST1*, *NF2* and *TAZ*, which are among the targets in the Hippo signaling pathway, were determined by quantitative real-time PCR. Figure 6 shows the expression profiles of PN Days 5, 10 and 15 groups for each gene. The absolute quantification method was used to analyze the dynamic process more precisely in the development of the PN period. The groups given sesame oil on behalf of the control group are symbolized by C, and the PN group given progesterone is symbolized by P. As seen in Fig. 6A, the expression of *YAP*, one of the main components of the Hippo signaling pathway, showed similar characteristics in the control and experimental groups on PN Day 5. *YAP* expression was observed at the highest level in the control group on PN Day 10, when gland development occurred. On the same day, it was determined that *YAP* expression was lower in the experimental group than in the control group. On PN Day 15, while *YAP* expression was observed in the control group, it was very low in the experimental group. *LATS1* expression, another essential component of the Hippo signaling pathway, showed very similar features to the expression of *YAP *(Fig. 6B). On PN Day 5, *LATS1* expression was similar in the control and experimental groups. On Day 10, *LATS1* expression was highest in the control group, while expression decreased in the experimental group. On PN Day 15, it was found that *LATS1* was lower in the experimental group. *LATS2* expression, shown in Fig. 6C, showed similar characteristics in the control and experimental groups on Day 5. On PN Days 10 and 15, *LATS2* expression was present in the control groups but decreased in the experimental groups. Figure 6D-F shows the altered expressions of *MST1*, *NF2* and *TAZ*, respectively. Similar profiles were obtained for these targets as well. Expressions on PN Day 5 did not change in the control and experimental groups. Expressions similarly decreased in the experimental groups on PN Days 10 and 15, except for *TAZ* on PN Day 10.

## Discussion

It has been stated that fertility is impaired in many animal models and can cause recurrent pregnancy loss, especially when the endometrium glands are absent [30]. Considering that the uterine glands, which secrete important substances, have critical importance for the implantation process, embryo or foetus survival and development [23, 24, 31]. Some studies show that conditional deletion of FOXA2 or CDH1, a cell–cell adhesion molecule, results in a marked reduction in the number of uterine glands [13, 14] or non-developed uterine glands in the neonatal uterus [16] In addition, deletion of Wnt7a and Wnt4 has been described as causing a reduced number of uterine glands or a non-gland appearance [8, 15]. Moreover, it was observed that uterine glands did not develop, and collagen accumulated in the stroma when FOXL2(Forkhead box protein L2) was overexpressed [32]. In our electron microscopy analysis, euchromatin nuclei, lipid droplets, and collagen synthesis in the stroma were among the similar features of the groups. Occasional separations in the epithelium and edematous areas were observed in the stroma of the experimental groups. These results may indicate that the use of progesterone to suppress the formation of glands during the experimental process particularly affects the cells in the stroma and may create wide gaps between the cells in the epithelium. This can indicate that communication between the epithelium and the stroma may be disrupted when the gland development inhibited.

In another study conducted on the proliferation of uterine epithelial cells, it was stated that, in the absence of CK1 alpha, mouse uterine adenogenesis was suppressed by the GSK3 beta pathway and the expression of FOXA2 by the p53 pathway, resulting in apoptosis in epithelial cells [7]. However, the mechanisms underlying the adenogenesis process in the PN period remain a mystery.

The Hippo signaling pathway has been shown to be very important at many stages in cell fate decisions, such as cell division, proliferation or death [18]. This signaling pathway has also been associated with many types of cancer [33]. At the same time, demonstration of the effectiveness of this signaling pathway in various diseases, such as cardiomyopathy and polycystic kidney disease, indicates that it may contribute to the development of treatment approaches [34, 35]. For example, when the Hippo signaling pathway is suppressed in the ovaries of patients with primary ovarian failure, the oocyte develops and successful pregnancy and delivery occur [36].

The key components of the Hippo signaling pathway, which works as a kinase cascade, are MST1/2, LATS1/2 and YAP/TAZ. Depending on whether MST1/2 and LATS1/2 are phosphorylated, YAP is located in the cytoplasm or in the nucleus. Phosphorylation of these proteins can be affected by many factors, such as the junctional units of the cells, the cytoskeleton or metabolism [18, 20]. The functions of the Hippo signaling pathway components and how they are active or inactive are still not fully known in many organs and tissues.

In the PN period, many dynamic events, such as the morphogenesis of the uterus and especially the differentiation, proliferation and maturation of cells during the gland development process, take place. In this study, our aim was to determine whether the Hippo signaling pathway is effective in the adenogenesis process that occurs in the postpartum uterus.

On PN Day 5, the control and experimental groups showed similar characteristics. YAP expression was observed in the epithelium and stroma. On PN Day 10, it was quite remarkable that the expression of YAP was high in both immunohistochemical, immunoblotting and RT-qPCR analyses in the control groups. Especially YAP expression was observed in the stroma and glands by immunohistochemical analysis. These data indicate that YAP can be highly effective, especially during adenogenesis and differentiation of cells. The YAP signal decreased in the experimental group on PN Day 10. We think that YAP, one of the key components of the Hippo signaling pathway, can play an effective role in uterine gland development. Considering the increase in the expression of YAP in decidual cells during pregnancy and its active efficacy in the implantation process [37], our findings in the uterine gland development process confirm that YAP is in mutual interaction with the development of the uterus glands. When compared to the control group, the higher expression was observed in the luminal epithelium in the experimental groups notably on the PN Day 10, indicates that there may be disruption in the communication between the epithelium and the stroma during gland development and may disturb the effectiveness of the Hippo signaling pathway. On PN Day 15, there was a decrease in YAP expression in the experimental group stroma cells. In the control group, the expression of YAP was lower on PN Day 15 than on PN Day 10. This result suggests that YAP undergoes a highly dynamic process in postpartum uterine morphogenesis.

p-YAP expression was observed in the stroma cells by the immunohistochemical method. Compared to the control groups, a weak uptake was observed in the stroma on Day 10 in gland development-impaired mice, while the signal in the luminal epithelium on Day 15 was different from the control group may indicate that when gland development is blocked, the Hippo signaling pathway may be disrupted and permanent damage may occur to the uterus. The p-YAP expression in the control groups had similar properties to YAP expression.

Another regulator of the Hippo signaling pathway is LATS1/2 which has a tumor suppressive property. Studies showing the effect of LATS1/2 on the uterus are quite limited [38]. First, it has been shown that uterine implantation defects can occur in mice in which Scribble, a protein that is important in cell polarisation and adhesion, was knocked out and LATS1 expression was decreased [39]. In another study, it was stated that this gene is also active in endometrial cancer [40]. It has been demonstrated that the conditional deletion of LATS1 and LATS2 causes the transformation of mouse Müllerian duct mesenchyme cells into myofibroblast cells and commonly causes developmental defects and sterility of reproductive system organs [41]. In our study, the gene expression of LATS1, in particular, showed very similar characteristics to the gene expression of YAP. The increase in expression in the control groups on PN Day 10 indicates that YAP may play an important role in the gland development process. PN Day 15 can be defined as the maturation process of the uterus in the postpartum period. The differences in gene expressions in the control groups compared to the experimental groups show that they can be effective in both the adenogenesis and maturation processes. On PN Days 10 and 15, it was observed that LATS2 expression decreased in the experimental groups compared to the control groups. Although LATS1/2 has been implicated as a component of the Hippo signaling pathway, it is important to evaluate LATS1 and LATS2 separately. LATS1-related proteins are associated more with estrogen signaling, and LATS2 is associated with cell cycle and metabolism [42]. These data reveal the effectiveness of LATS1 and LATS2 in PN changes in the uterus and suggest that they can affect the Hippo signaling pathway through different mechanisms.

Studies on MST1 and MST2 show that these genes are effective in maintaining the size of organs and in tumor development [43, 44]. The deletion of MST1 and MST2 in the epididymis, mice were infertile [45]. This study also presents the view that the Hippo signaling pathway may also be effective for infertility. In our study, we observed differences in MST1 gene expression in the experimental and control groups on PN Days 10 and 15. The fact that MST1 was at the highest level in the control groups, especially on Day 15, which is the maturation period, indicates that it may be more effective in the maturation process.

It was noted that when supressing the Merlin protein (NF2), which is an activator of the Hippo signaling pathway and an important suppressor of YAP/TAZ [46] is involved in polarisation in many epithelial types and also in the maturation of apical junctional units [47], uterine gland development does not occur [48]. In our study, it was shown that NF2 expression was effective on PN Days 10 and 15 in the control groups but decreased in the experimental groups. These data also show that NF2 may be effective in uterine morphogenesis during adenogenesis and maturation.

Studies on TAZ, a transcriptional coactivator containing a PDZ-related motif that is a paralog of YAP and also known as WWTR [49] have revealed that TAZ is necessary for spermatogenesis and fertility [50]. It is activated in human stromal cells [51] and plays an active role in decidualization, directing stromal cells to proliferation in experimental animals [52]. In our study, although it was observed that this signal decreased in the experimental groups during the adenogenesis and maturation processes, a significant difference appeared in the maturation process. TAZ expression was also compatible with the Hippo signaling pathway-related gene expressions that we investigated. This means that the Hippo signaling pathway can be active in the process of gland development, differentiation and maturation.

In conclusion, the activity of YAP, which is the main component of the Hippo signaling pathway, was clearly demonstrated by immunohistochemical, immunoblotting and RT-qPCR analyses on PN Day 10, which is a very important day for the development of the uterine gland, and on PN Day 15, when the maturation process occurs in our study. During this period, it was determined that YAP expression declined in mice with inhibited uterine gland development. These results show that YAP can play an active role in gland development and the maturation process. The location of both YAP and p-YAP, especially in the cytoplasm of the epithelium, stroma and gland epithelium, indicates that the Hippo signaling pathway is active during these periods. Other important components of the Hippo signaling pathway, LATS1, LATS2 and MST1, also show a profile consistent with these results. Moreover, NF2, which affects the Hippo signaling pathway, is also seen in lower levels in mice with inhibited gland development, indicating that the Hippo signaling pathway may be effective in the uterus during the PN period. In addition, on the PN day of 15, which is the maturation process of the mouse uterus, the difference between the control and experimental groups about TAZ can shows that the Hippo signaling pathway may have other effects on different days of uterine development.

Uterine glands play a very important participatory role in the implantation and pregnancy processes. Mechanisms related to uterine gland development may underlie recurrent miscarriages and failed implantation. At the same time, considering that the glands play an active role in the development of cancer in the uterus, understanding the mechanisms in the gland development process may be important in treating diseases such as infertility and cancer. In this study, in particular, we observed that the expression of Hippo signaling pathway-related components decreased when gland development was inhibited. These data also show that the Hippo signaling pathway can be associated with infertility.

## Supplementary Information

Below is the link to the electronic supplementary material.

Figure S1 Postnatal uterus development stages (**A**) and Hippo signaling pathway components (**B**) (PNG 888 KB)

High resolution image (TIF 718 kb)

Figure S2 Experimental setup of our study (PNG 858 KB)

High resolution image (TIF 512 kb)

Figure S3 Negative control staining of YAP. Postnatal 5 Day of Control (**A**,**B**) and Experimental Groups (**C**,**D**). **B** and **D** were negative control stainings (PNG 5.57 MB)

High resolution image (TIF 11.8 mb)

Figure S4 Negative control stainings of p-YAP. Postnatal 5 Day of Control (**A**,**B**) and Experimental Groups (**C**,**D**). **B** and **D** were negative control stainings (PNG 5.99 MB)

High resolution image (TIF 12.8 mb)

Supplementary file1 (PDF 383 kb)

Supplementary file2 (PDF 199 kb)

Supplementary file3 (DOCX 33.3 kb)

Supplementary file5 (DOCX 14.4 kb)

## Data Availability

The data underlying this article will be shared on reasonable request to the corresponding author.
