# Supplementary figures and images for: Alterations in the Hippo Signaling Pathway During Adenogenesis Impairment in Postnatal Mouse Uterus
Source: Reprod Sci. 2025 Feb 11;32(5):1685–98. doi: 10.1007/s43032-025-01793-y (PMC12041100; doi:10.1007/s43032-025-01793-y)

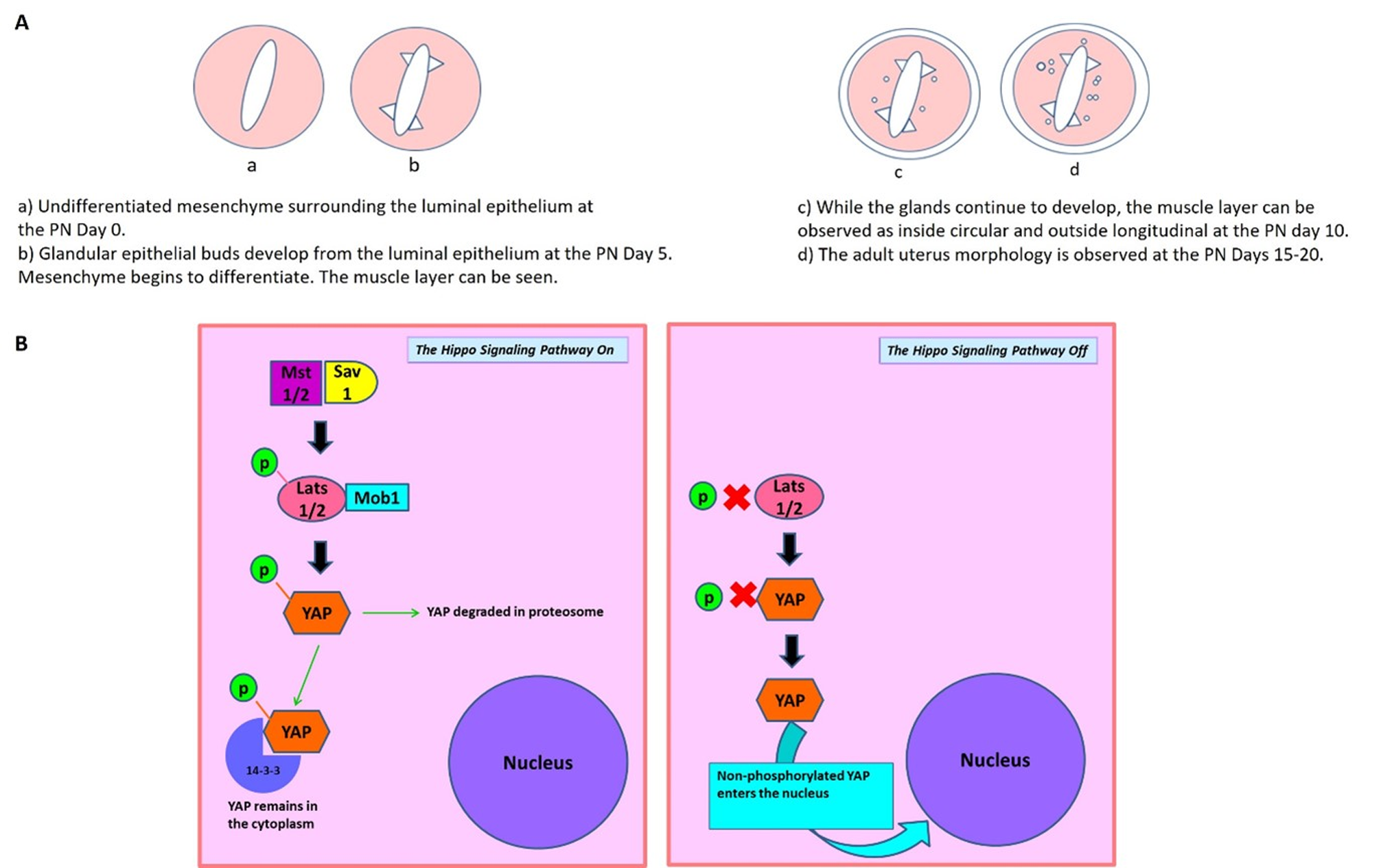

Supplement: Supplementary file 1 — Postnatal uterus development stages (A) and Hippo signaling pathway components (B) (PNG 888 KB) [file 43032_2025_1793_Fig7_ESM.png]

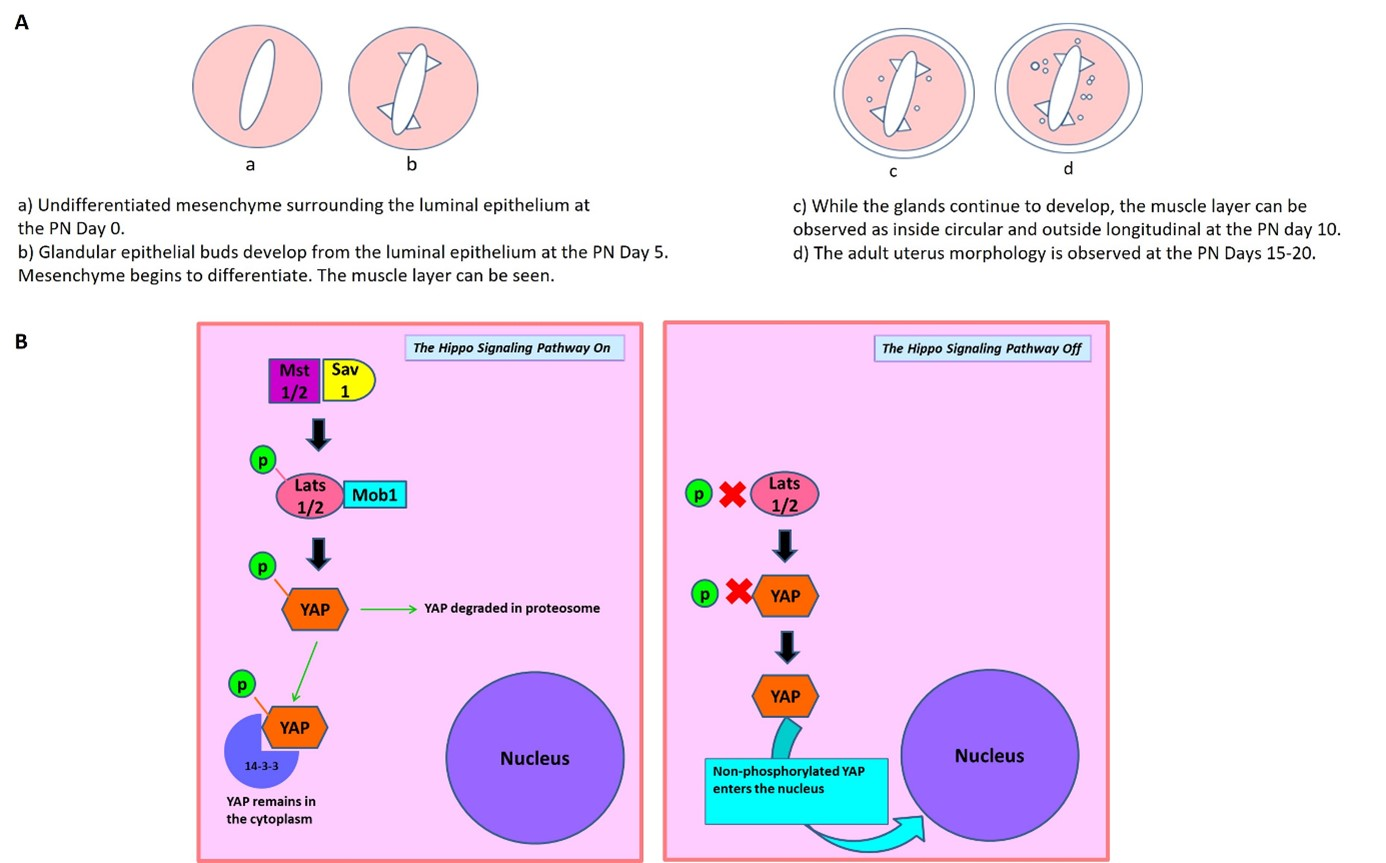

Supplement: Supplementary file 2 — High resolution image (TIF 718 kb) [file 43032_2025_1793_MOESM1_ESM.tif]

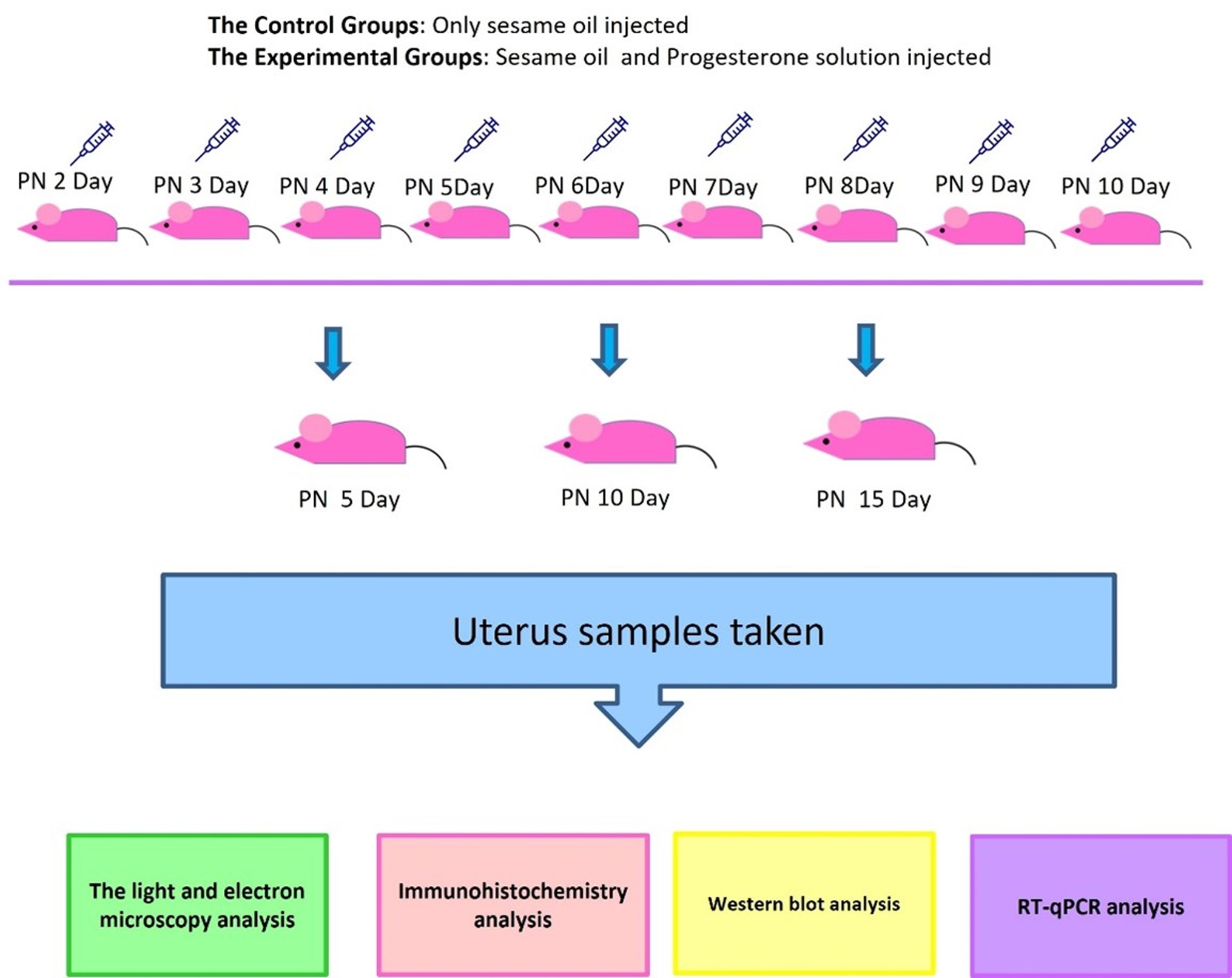

Supplement: Supplementary file 3 — Experimental setup of our study (PNG 858 KB) [file 43032_2025_1793_Fig8_ESM.png]

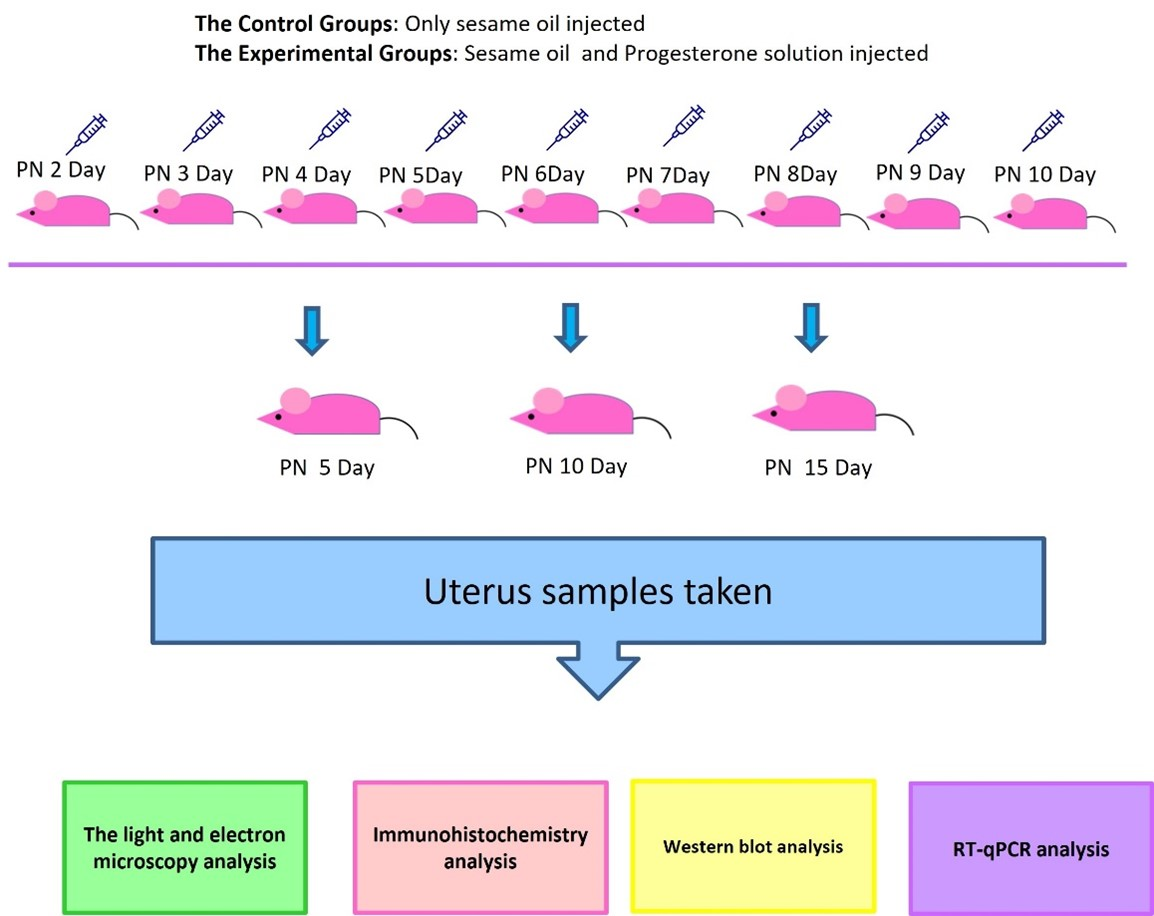

Supplement: Supplementary file 4 — High resolution image (TIF 512 kb) [file 43032_2025_1793_MOESM2_ESM.tif]

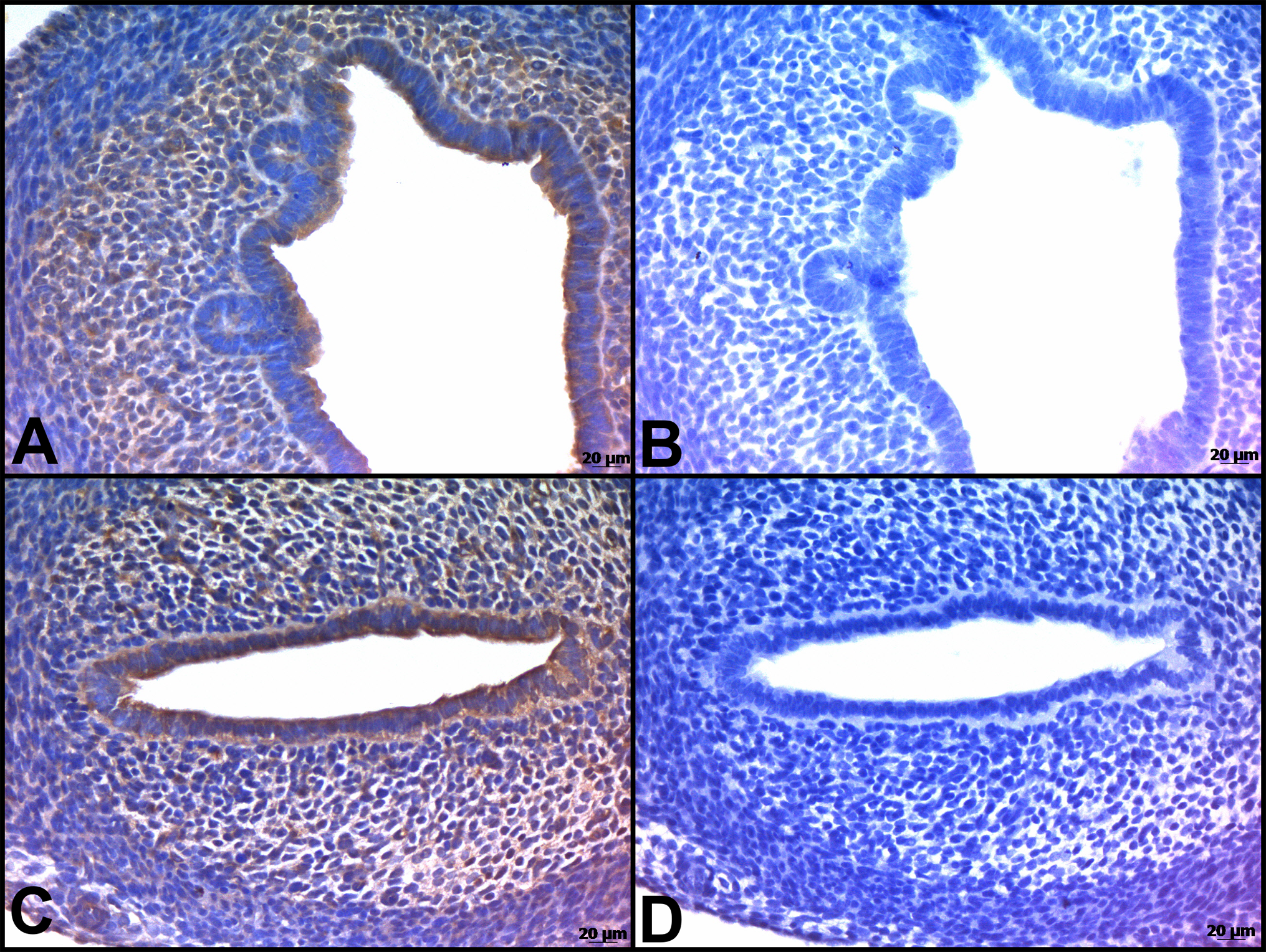

Supplement: Supplementary file 5 — Negative control staining of YAP. Postnatal 5 Day of Control (A,B) and Experimental Groups (C,D). B and D were negative control stainings (PNG 5.57 MB) [file 43032_2025_1793_Fig9_ESM.png]

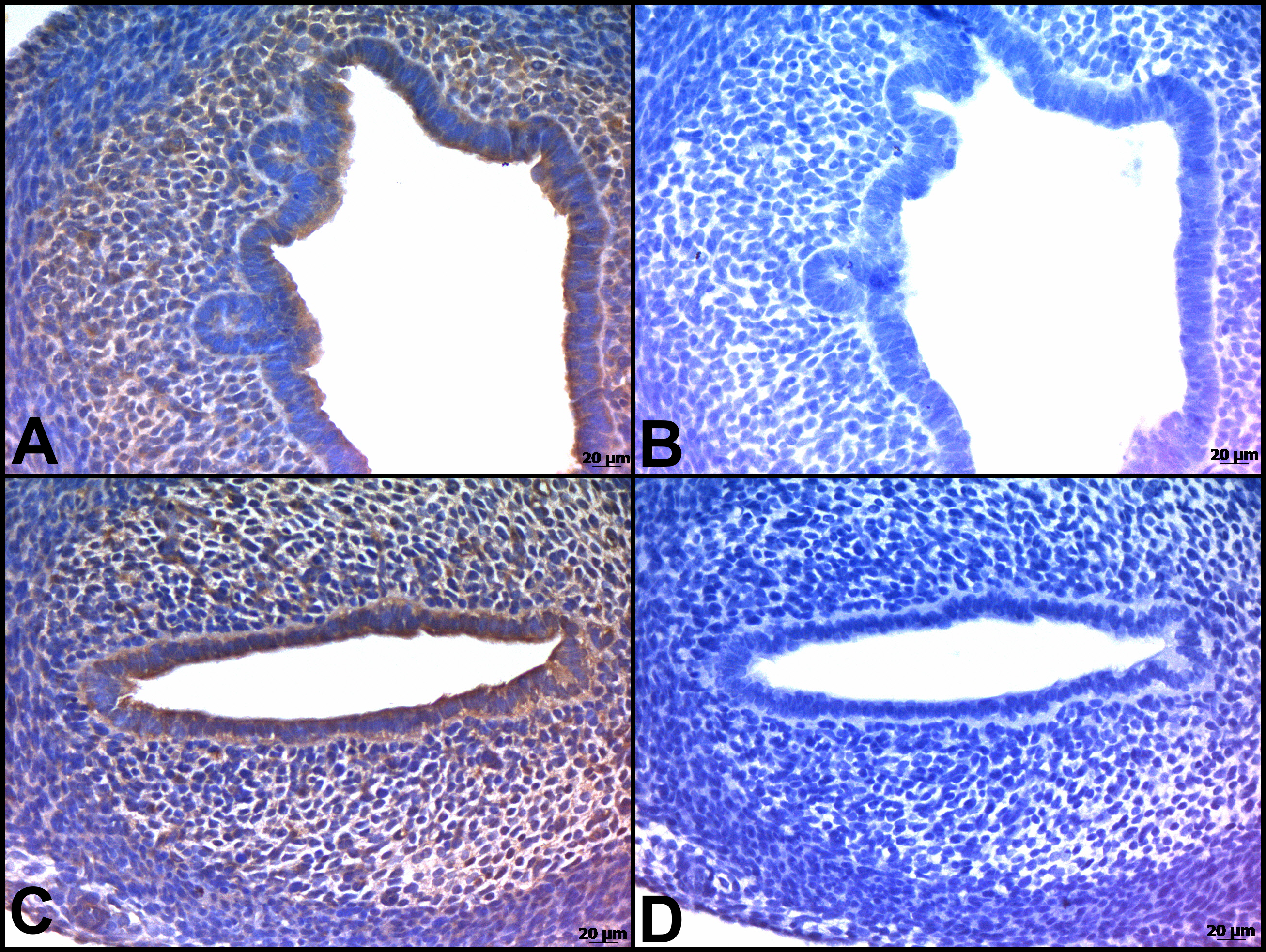

Supplement: Supplementary file 6 — High resolution image (TIF 11.8 mb) [file 43032_2025_1793_MOESM3_ESM.tif]

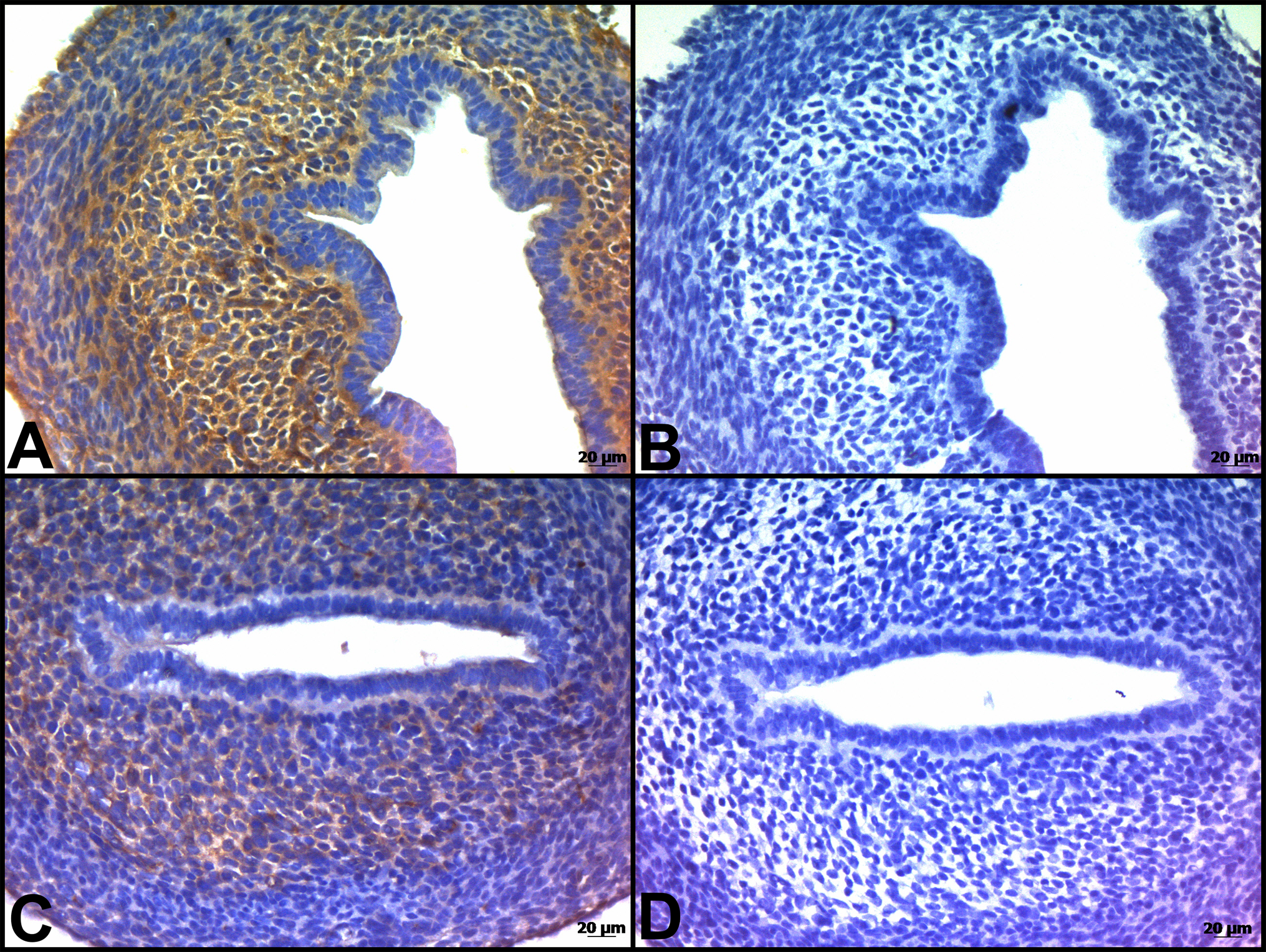

Supplement: Supplementary file 7 — Negative control stainings of p-YAP. Postnatal 5 Day of Control (A,B) and Experimental Groups (C,D). B and D were negative control stainings (PNG 5.99 MB) [file 43032_2025_1793_Fig10_ESM.png]

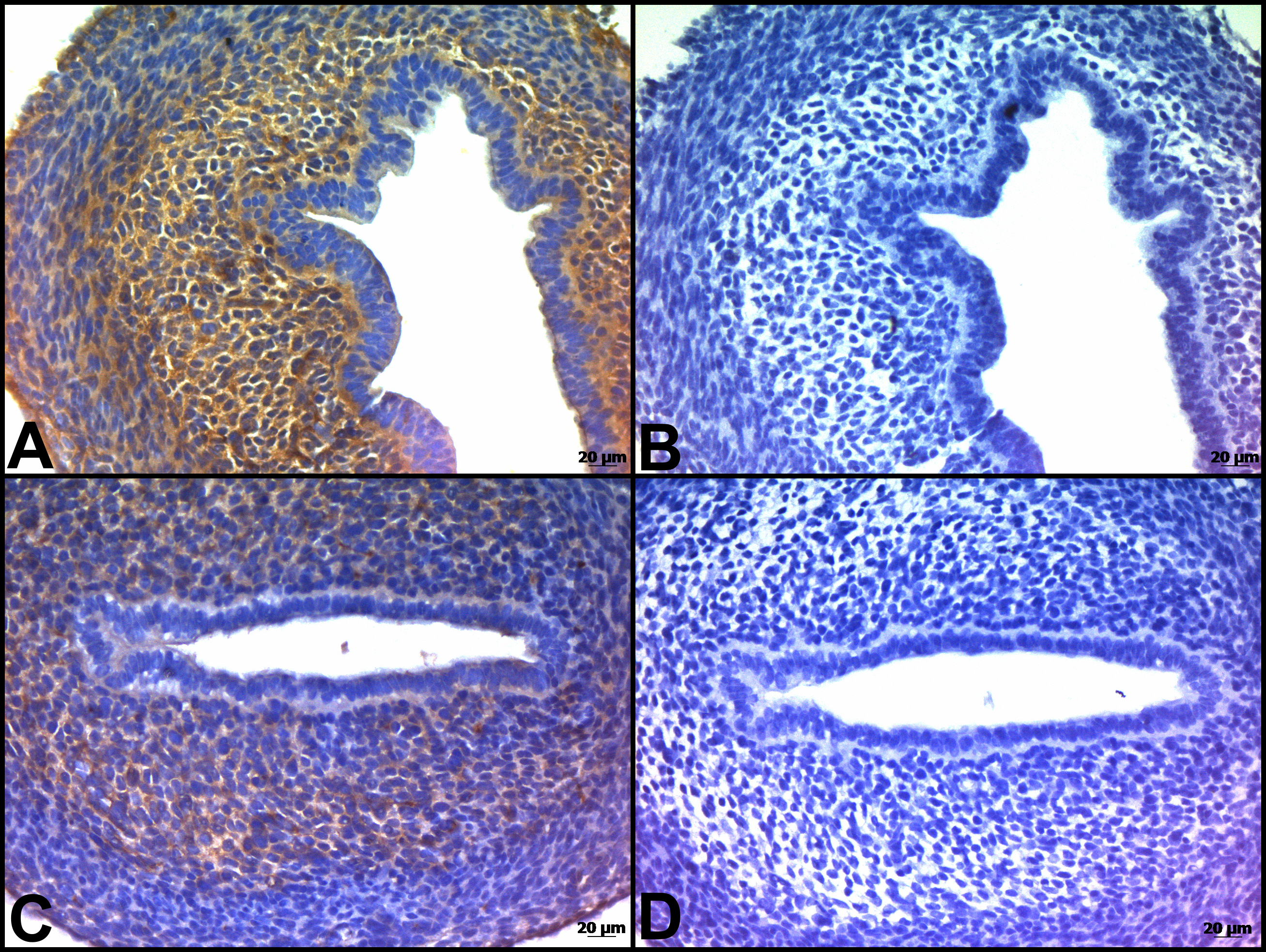

Supplement: Supplementary file 8 — High resolution image (TIF 12.8 mb) [file 43032_2025_1793_MOESM4_ESM.tif]
