# Supplementary material for: Alterations in the Hippo Signaling Pathway During Adenogenesis Impairment in Postnatal Mouse Uterus
Source: Reprod Sci. 2025 Feb 11;32(5):1685–98. doi: 10.1007/s43032-025-01793-y (PMC12041100; doi:10.1007/s43032-025-01793-y)
Supplement: Supplementary file 9 — (PDF 383 kb) [file 43032_2025_1793_MOESM5_ESM.pdf]

T.C.
ANKARA ÜNİVERSİTESİ REKTÖRLÜĞÜ
Hayvan Deneyleri Yerel Etik Kurulu

HAYVAN DENEYLERİ YEREL ETİK KURULU KARARI

TOPLANTI TARİHİ : 23/12/2020
TOPLANTI NO : 2020-21
DOSYA NO : 2020-136
KARAR NO : 2020-21-168

Yürütücülüğünü Üniversitemiz Tıp Fakültesi, Histoloji ve Embriyoloji Anabilim Dalı öğretim üyelerinden Prof.Dr.Esra Erdemli'nin yaptığı, araştırıcı olarak Doç.Dr.Arzu Atalay ve Araş.Gör. İrem İnanç'ın katıldığı "Uterus bez gelişimi engellenmiş farelerde Hippo sinyal yolağı bileşenlerinden YAP ve p-YAP etkinliğinin belirlenmesi" başlıklı çalışma Kurulumuzca değerlendirilmiş ve söz konusu çalışmanın Üniversite senatosunun 12/2/2016 tarihli toplantısında 430/3642 sayılı kararı ile kabul edilen ve Hayvan Deneyleri Merkezi Etik Kurulu'nun 19/2/2016 tarih ve 42 sayılı kararı ile onaylanan "Ankara Üniversitesi Hayvan Deneyleri Yerel Etik Kurulu Yönergesi" ne göre aşağıda belirtilen kapsamda yapılmasına oy birliği ile karar verilmiştir.

Hayvan Türü : Fare
Hayvan Sayısı : 40
Geçerlilik Süresi : 14/06/2021-14/06/2022

ETİK KURUL ÜYELERİ

Unvanı / Adı / Soyadı	Uzmanlık Dalı	Kurumu	Cinsiyeti	İmza
Prof. Dr. M. Taner KARAOĞLU (Başkan)	Viroloji Anabilim Dalı	Veteriner Fakültesi	E	
Prof. Dr. Tanju ÖZÇELİKAY (Başkan Vekili)	Farmakoloji Anabilim Dalı	Eczacılık Fakültesi	E	
Prof. Dr. Emine DEMİREL YILMAZ (Üye)	Tıbbi Farmakoloji Anabilim Dalı	Tıp Fakültesi	K	
Prof. Dr. Nuri YİĞİT (Üye)	Zooloji Anabilim Dalı	Fen Fakültesi	E	
Prof. Dr. Fatin CEDDEN (Üye)	Hayvan Yetiştirme Anabilim Dalı	Ziraat Fakültesi	E	
Prof. Dr. Mine KIRKAĞAÇ (Üye)	Su Ürünleri Mühendisliği Bölümü	Ziraat Fakültesi	K	

Adres: Ankara Üniversitesi Rektörlüğü 06100 - Tandoğan / ANKARA **Tel:** 0 (312) 212 60 40 / 2101 **Faks:** 0 (312) 212 60 49
