# Supplementary material for: Alterations in the Hippo Signaling Pathway During Adenogenesis Impairment in Postnatal Mouse Uterus
Source: Reprod Sci. 2025 Feb 11;32(5):1685–98. doi: 10.1007/s43032-025-01793-y (PMC12041100; doi:10.1007/s43032-025-01793-y)
Supplement: Supplementary file 10 — (PDF 199 kb) [file 43032_2025_1793_MOESM6_ESM.pdf]

Doç. Dr. Gülnur GÖLLÜ BAHADIR (Üye)	Çocuk Cerrahisi Anabilim Dalı	Tıp Fakültesi	K	
Doç. Dr. Halit KANCA (Üye)	Doğum ve Jinekoloji Anabilim Dalı	Veteriner Fakültesi	E	
Dr. Vet. Hek. Nigar YERLİKAYA (Üye)	Veteriner Hekimliği Tarihi ve Deontoloji Anabilim Dalı	Veteriner Fakültesi	K	
Dr. Vet. Hek. Gürbüz ERTÜRK (Üye)	Active Veteriner Sağlık Merkezi	Serbest	E	
Uzm. Vet. Hek. Hüseyin DEDE (Üye)	Veteriner Hekimler Derneği	Serbest	E	
Uzm. Vet. Hek. Attila İŞGÖREN (Üye)	Deney Hayvanları Yetiştirme ve Araştırma Laboratuvarı	Tıp Fakültesi	E	
Fatma Aysun COŞKUN (Üye)	İktisat	Serbest	K	
