# Supplementary material for: Alterations in the Hippo Signaling Pathway During Adenogenesis Impairment in Postnatal Mouse Uterus
Source: Reprod Sci. 2025 Feb 11;32(5):1685–98. doi: 10.1007/s43032-025-01793-y (PMC12041100; doi:10.1007/s43032-025-01793-y)
Supplement: Supplementary file 11 — (DOCX 33.3 kb) [file 43032_2025_1793_MOESM7_ESM.docx]

| 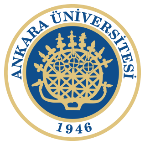 | **REPUBLIC OF TURKEY**  **ANKARA UNIVERSITY RECTORATE**  **Animal Tests Local Ethics Commission**  **RESOLUTION OF ANIMAL TESTS LOCAL ETHICS COMMITTEE** |
| --- | --- |

**MEETING DATE** : 23/12/2020

**MEETING NO** : 2020 -21

**FILE NO** : 2020 – 136

**RESOLUTION NO** : 2020 -21-168

The study titled “Identification of the activity of YAP and p-YAP, components of the Hippo signaling pathway in mice with impaired uterine gland development” conducted by Prof. Dr. Esra Erdemli, a faculty member of the Department of Histology and Embryology, Faculty of Medicine of our University, and with the attendance of Assoc. Prof. Dr. Arzu Atalay and Res. Asst. İrem İnanç as researchers has been assessed by our Commission and it was unanimously resolved that the study in question would be conducted within the scope specified below, in accordance with "Ankara University Animal Tests Local Ethics Committee Directive", which was Adopted by the resolution numbered 430/3642 of the University Senate at its meeting dated 12/2/2016 and approved by the Animal Tests Center Ethics Committee with the resolution numbered 42 dated 19/2/2016.

Animal Type : Mice

Number of Animals : 40

Validity Period : 14/06/2021 – 14/06/2022

| ETHICS COMMITTEE MEMBERS | | | | |
| --- | --- | --- | --- | --- |
| Title / Name / Surname | Major Branch | Institute | Gender | Signature |
| Prof. Dr. M. Taner KARAOĞLU (Chairman) | Department of Virology | Faculty of Veterinary Medicine | M |  |
| Prof. Dr. Tanju ÖZÇELİKAY (Acting Chairman) | Department of Pharmacology | Faculty of Pharmacy | M |  |
| Prof. Dr. Emine DEMİREL YILMAZ (Member) | Department of Medical Pharmacology | Medical Faculty | F |  |
| Prof. Dr. Nuri YİĞİT (Member) | Department of Zoology | Faculty of Science | M |  |
| Prof. Dr. Fatin CEDDEN (Member) | Department of Animal Husbandry | Faculty of Agriculture | M |  |
| Prof. Dr. Mine KIRKAĞAÇ (Member) | Department of Aquacultural Engineering | Faculty of Agriculture | F |  |
