# Supplementary material for: Alterations in the Hippo Signaling Pathway During Adenogenesis Impairment in Postnatal Mouse Uterus
Source: Reprod Sci. 2025 Feb 11;32(5):1685–98. doi: 10.1007/s43032-025-01793-y (PMC12041100; doi:10.1007/s43032-025-01793-y)
Supplement: Supplementary file 12 — (DOCX 14.4 kb) [file 43032_2025_1793_MOESM8_ESM.docx]

| Assoc. Prof. Dr. Gülnur GÖLLÜ BAHADIR (Member) | Department of Pediatric Surgery | Medical Faculty | F |  |
| --- | --- | --- | --- | --- |
| Assoc. Prof. Dr. Halil KANCA (Member) | Department of Obstetrics and Gynecology | Faculty of Veterinary Medicine | M |  |
| Dr. Vet. Physician Nigar YERLİKAYA (Member) | Department of Veterinary History and Deontology | Faculty of Veterinary Medicine | F |  |
| Dr. Vet. Physician Gürbüz ERTÜRK (Member) | Active Veterinary Health Center | Independent | M |  |
| Specialist Vet. Physician Hüseyin DEDE (Member) | Veterinary Medical Association | Independent | M |  |
| Specialist Vet. Physician Attila İŞ GÖREN (Member) | Laboratory of Test Animals Breeding and Research | Medical Faculty | M |  |
| Fatma Aysun COŞKUN '(Member) | Economics | Independent | F |  |
